# Study on the anti-biofilm mechanism of 1,8-cineole against *Fusarium solani* species complex

**DOI:** 10.3389/fphar.2022.1010593

**Published:** 2022-10-14

**Authors:** Yu Zhang, Yiming Wang, Xinghong Zhao, Lu Liu, Rui Xing, Xu Song, Yuanfeng Zou, Lixia Li, Hongping Wan, Renyong Jia, Lizi Yin, Xiaoxia Liang, Changliang He, Qin Wei, Zhongqiong Yin

**Affiliations:** ^1^ Natural Medicine Research Center, College of Veterinary Medicine, Sichuan Agricultural University, Chengdu, China; ^2^ Key Laboratory of Animal Disease and Human Health of Sichuan Province, Sichuan Agricultural University, Chengdu, China; ^3^ Yibin university Sichuan Oil Cinnamon Engineering Technology Research Center, Yibin University, Yibin, China

**Keywords:** *Fusarium solani* species complex, biofilm, 1, 8-cineole, adhesion, mitochondrial, transcriptome sequencing

## Abstract

Fungal-infections are mostly due to fungi in an adhering, biofilm-mode of growth and not due to planktonically growing, suspended-fungi. 1, 8-cineole is a natural product, which has been shown to possess antifungal effect. However, the anti-biofilm effect and mechanism of 1,8-cineole against *Fusarium solani* species complex has not reported previously. In this study, we found that 1,8-cineole has a good antifungal activity against *F. solani* with an MIC value of 46.1 μg/ml. Notably, 1,8-cineole showed good anti-biofilm formation activity against *F. solani via* inhibiting cell adhesion, hypha formation and decreasing the secretion of extracellular matrix at the concentration of ≥5.76 μg/ml. In addition, transcriptome sequencing analysis results showed that *F. solani* species complex genes related to ECM, protein synthesis and energy metabolism were down-expressed in the biofilms formation process treated with 1,8-cineole. In conclusion, these results show that 1,8-cineole has good anti-biofilm formation activity against *F. solani* species complex, and it exerts its anti-biofilm formation activity by downregulating of ergosterol biosynthetic genes, inhibiting adhesion, hindering the synthesis of ECM and interfering mitochondrial activity. This study suggests that 1,8-cineole is a promising anti-biofilm agent against *F. solani* species complex.

## 1 Introduction

*Fusarium spp*. can cause infections not only in plants but also in humans and animals, especially members of the *Fusarium oxysporum* and *F. solani* species complex, which have been implicated in human infections worldwide (Dalyan Cilo et al., 2015; van Diepeningen and de Hoog, 2016). The number of human infections by *Fusarium spp*. is on the rise worldwide due to an increase in the number of susceptible and immunocompromised people (Batista et al., 2020). Studies showed that up to 10% of onychomycosis is caused by *Fusarium spp*. (van Diepeningen et al., 2015). Eye infections associated with plants or soil commonly resulted in keratomycosis. Moreover, it could cause life-threatening infections in patients with severe immunodeficiency, especially leukaemia (van Diepeningen et al., 2015). The biofilm of filamentous fungi is one of its pathogenic factors. Infections associated with biofilm formation have been considered as a significant and increasing clinical problem.

Fungal biofilm is a community of microorganisms that are embedded in a polymeric matrix attached to an object’s surface. Biofilm formation is a very complicated process involving initial adhesion, hyphae formation, maturation and dispersion (Liu et al., 2021). It has been reported that a dramatic change will happen in biofilms of filamentous fungi from planktonic organisms to cells, such as the metabolism and biosynthesis (O’Toole et al., 2000). The extracellular matrix (ECM) of biofilm normally includes proteins, polysaccharides, lipids, and extracellular DNA (eDNA) (Chiang et al., 2013), which is one of the main protective component of microbial biofilms.

Although there are many kinds of antifungal drugs for treating fusariosis, such as amphotericin B and voriconazole, the cure rate is low in clinical trials (Guarro, 2013). Moreover, these drugs have serious toxicity concerns, such as voriconazole can increase risk of cutaneous malignancies (Levine Miriam, T., and Chandrasekar Pranatharthi, H. (2016))and amphotericin B is related to nephrotoxicity (Hamill, 2013). Therefore, it is urgently needed to discover novel and effective medicines to treat fusariosis. Natural products from plants are one of the important resources for drug discovery. 1,8-cineole, a kind of cyclic-ether monoterpene, which is widely exist in plants, including Eucalyptus, tea, cinnamon, bay and sage (Rodenak-Kladniew et al., 2020; Cai et al., 2021). 1,8-cineole has been shown with multiple biological activities in the treatment of cancers (Sampath et al., 2018), neuropathic pain (Zhang et al., 2018), cardiovascular illnesses (Wang et al., 2021), digestive disorders (Santos et al., 2004), nervous system disease (Figuêiredo et al., 2019) and antimicrobial (De Sousa et al., 2012). However, its activity against *Fusarium solani* species complex biofilms has not yet been intensively investigated.

In this study, we investigated the anti-biofilm formation activity of 1,8-cineole against *F. solani*. Moreover, the effect of 1,8-cineole on *F. solani* species complex biofilm architecture was visualized using optical microscopy, scanning electron microscopy (SEM), and confocal laser scanning microscopy (CLSM). Furthermore, to investigate the influence of 1,8-cineole on the biofilm formation of *F. solani* at the gene expression level, transcriptome sequencing analysis was performed on the *F. solani* biofilm treated with 1,8-cineole. To the best of our knowledge, this is the first study to estimate the anti-biofilm formation activity of 1,8-cineole against *F. solani*, and also the first time RNA-seq analysis was used to explore the anti-biofilm formation mechanism of 1,8-cineole.

## 2 Materials and methods

A clinical strain of *Fusarium solani* species complex preserved by Sichuan Agricultural University was used in this study. Frozen stock of the strain was revived by subculture on sabouraud dextrose agar (SDA; Qingdao Hope Bio-Technology Co., Ltd. Qingdao, China) plates at 27°C for 4–6 days, and the conidia were subsequently harvested in PBS (0.02 M phosphate, 0.15 M, pH 7.2). The resulting *F. solani* suspensions counted on a hemocytometer. For all experiments, the final sporangiospore concentration was 2 × 10^6^ CFU/ml planktonic cells. 1,8-cineole was dissolved in 40% acetone (XILONG SCIENTIFIC CO., LTD. Sichuan, China) to a concentration of 461 μg/ml, and 1,8-cineole was further diluted to 92.2 μg/ml in RPMI 1640 medium (Hyclone, Logan, UT, United States) and used to prepare a series of 2-fold dilutions ranging from 2.88 to 92.2 μg/ml.

### 2.1 Antifungal susceptibility testing

The minimal inhibitory concentrations (MICs) of 1,8-cineole were determined by the microdilution method using the approach of the Clinical and Laboratory Standards Institute (CLSI) guideline in 96-well microplates (Fothergill, 2012). 100 μl of *F. solani* species complex suspension (2 × 10^6^ CFU/ml) and 100 μl of 1,8-cineole (in the range of 92.2 to 2.88 μg/ml in the above described medium) were added in each well and incubated at 27°C for 24 h. Wells containing only 40% acetone served as controls, and wells without test compounds served as controls blank control. To determine the minimal fungicidal concentrations (MFC), fungal cells from MIC assays were collected and rinsed with fresh RPMI 1640 medium, and inoculated on SDA plates. Colonies were counted after 3 days of incubation. The MFC was defined as the lowest concentration of testing compound at which 99.9% of the microorganisms were killed.

### 2.2 *In vitro* biofilm inhibition assay

The biofilm formation and the viability of the cells were tested after 1,8-cineole treatments (Zhong et al., 2017). Briefly, 100 μl *F solani* species complex suspensions (2 × 10^6^ CFU/ml) were added to a 96-well microplate to adhesion for 90 min at 27°C. After pre adhesion, the unattached cells were washed by using sterile PBS, then add 100 μl 1,8-cineole (in the range of 23.05 to 2.88 μg/ml in the above described medium), and incubated at 27°C for 72 h to evaluate the effect of 1,8-cineole on biofilm formation.

The inhibitory effect of 1,8-cineole on *F. solani* species complex biofilm was evaluated using the 2,3-bis-(2-methoxy-4-nitro-5-sulfophenyl)-2H-tetrazolium-5-carboxanilide (XTT) (Shanghai yuanye Bi-Technology Co., Ltd. Shanghai, China) reduction assay according to the method described in a previous study (Liu et al., 2017). After incubation at 27 C for 72 h, the biofilms were washed twice with PBS to remove unattached cells and 200 μl XTT solution containing 1 mM menadione was added. After incubation at 37 C for 2 h in dark, 100 μl of each supernatant were transferred to new 96-well plates and the absorbance of each solution was measured at 490 nm using SPECTROstar Nano plate reader (BMG LabTech, Offenburg, Germany).

### 2.3 Microscopic observations on biofilms

Three microscopic techniques were used to investigate the structure of biofilms: optical microscopy, SEM, and CLSM. Incubation of *F. solani* cells and 1,8-cineole for 72 h without shake, to observe the effect on biofilms, and the final concentrations of 1,8-cineole were adjusted to 5.76, 11.52, 23.05 μg/ml, respectively.

The expolysaccharides was detected using the argentation method as described in a previous study (Bai and Cui, 2019). Briefly, put the coverslips containing biofilms into 2.5% glutaraldehyde solution and fixation for 2 h, and then, saturated calcium chloride solution was added to coverslips for 15 min 5% silver nitrate solution was added to coverslips and incubated for 15 min. After that, the biofilm was treated with 1% hydroquinone solution for 2 min, and washed with sterilized water for three times. The coverslips containing biofilms were placed in 5% sodium thiosulfate for 2 min, and washed with sterilized water for three times. Finally, the samples were dried and observed under an optical microscope for photography.

For SEM analysis, biofilms were washed and placed in 2.5% glutaraldehyde at 4°C overnight to fix samples (Wu et al., 2020). Following, the samples were dehydrated with ethanol in ascending concentrations (30, 50, 70, 80, 90, 95, and 100% ethanol) for 10 min at each concentration, and dried overnight. After drying, the samples were covered with a gold layer and observed with an Inspect FEI 50 scanning electron microscope at 20 kV. The images were processed by Photoshop software (Adobe Systems, San Jose, CA, United States).

For CLSM analysis, the coverslips containing biofilms were washed with sterile PBS, and the biofilms were stained with the Live/Dead TM viability/Cytotoxicity Assay Kit to distinguish live and dead cells. NucGreen is a green fluorescent dye and viable cells with intact membranes were stained green, while EthD-Ⅲ is a red fluorescent dye, which is not permeable by the viable cell membrane, and its fluorescence during microscopy studies indicates the cell death. The biofilms were visualized by STELLARIS STED/EM CPD 300 confocal laser scanning microscopy (Brilhante et al., 2019; Xie et al., 2020).

### 2.4 Adhesion assay

100 μl *F solani* species complex suspensions and 100 μl 1,8-cineole (in the range of 23.05 to 2.88 μg/ml in the above described medium) were added and incubated at 96-well microplates at 27°C for 4 h. Following the initial incubation, the medium was aspirated and unattached cells were washed by using sterile PBS thrice. Adhesion assay was measured by XTT-reduction assay as described previously (Zhong et al., 2017). The colorimetric change at 490 nm was measured with a SPECTROstar Nano plate reader (BMG LabTech, Offenburg, Germany).

### 2.5 Hyphae growth inhibition assay

In a 6-well microplate, 1 ml cell suspension (2 × 10^6^ CFU/ml) was treated with different concentrations of 1,8-cineole (in the range of 2.88–23.05 μg/ml in the above described medium) and incubated at 27°C for 6 h. After 6 h of incubation, the culture plate was washed three times with sterile PBS, and then all samples were visualized under bright field using inverted microscope and photographed.

### 2.6 Composition of *F. solani* species complex biofilm and biomass quantification

1 ml cell suspension (2 × 10^6^ CFU/ml) and 1 ml 1,8-cineole (in the range of 46.1 to 11.52 μg/ml) were added in 6-well microplates and incubated in RPMI 1640 medium 27°C for 72 h. Firstly, the contents of extracellular protein, extracellular polysaccharide and eDNA in biofilm of *F. solani* species complex were determined by enzymatic hydrolysis method. The samples were treated with protease K (100 μg/ml), DNaseI (2 mg/ml) or sodium periodate (10 μM) at 27°C for 2 h. After that, the biofilm was stained with crystal violet (CV), decolorized with 33% glacial acetic acid, and the OD_595_ value was determined with a SPECTROstar Nano plate reader (Sacks et al., 2018).

The extraction of ECM was performed as the method described in a previous study (Gupta et al., 2021). The biofilm was treated with 1,8-cineole in 6-well plates according to the above method. After that, the wells were washed to remove the media and 1,8-cineole and the biofilm was scrapped in PBS. The biofilm was sonicated at 35W for five cycles (30 s each) on ice bath and then the suspension was centrifuged for 5 min at 12,000 rpm. The cell pellet was visualized under light microscope for any cell lysis or breakage. The supernatant was used for extracelluar protein, extracellular polysaccharide (EPS), eDNA.

The EPS in *F. solani* species complex biofilm was estimated by a congored binding assay. The Congo red was added to 1 ml suspension to a final concentration of 40 μg/ml, and incubated at 37°C for 2 h at 200 r/min in a constant temperature shaker. Then the incubated solution was centrifuged at 5,000 g for 5 min to precipitate the cells, and the absorbance of supernatant was measured at 490 nm (Desai et al., 2019).

The Bradford method was used to estimate the protein content in the biofilm. According to manufacturer’s instructions, the protein concentration of samples can obtain by standard protein curves.

The eDNA was estimated by phenol-chloroform method. Briefly, The DNA of the ECM fraction was extracted with an equal volume of phenol: trichloromethane: isoamyl alcohol (25:24:1, v/v) (Invitrogen, Carlsbad, CA, United States) and then precipitated with trichloromethane: isoamyl alcohol (24:1). After centrifugation, 1/10 equivalent (v/v) of 3M sodium acetate and 2.5 equivalent (v/v) of anhydrous ethanol were added to the separated upper water layer and stored overnight at −20°C. The next day, the solution was centrifuged at 12,000×g for 10 min to obtain the precipitate, the precipitate was dissolved in 100 μl sterile distilled water, and the absorbance was measured at 260 nm.

### 2.7 Transcriptomic analysis

*F. solani* species complex was incubated in RPMI-1640 with or without 5.76 μg/ml of 1,8-cineole (MIC = 46.1 μg/ml) for 72 h. After removing the planktonic cells, the biofilms were collected and subsequently frozen in liquid nitrogen. After mRNA purification and sequencing library construction, the samples were sequenced by next-generation sequencing (NGS) based on the illumine platform. The threshold value of DEGs (differential expression genes) is a fold change of >2 and a *p* value of <0.05. RNA extraction, mRNA purification, and cDNA synthesis and sequencing were performed by Beijing Novogene (China). Three independent biological replicates were performed for each treatment. The RNA-Seq data have been deposited in the NCBI Gene Expression Omnibus with the accession number PRJNA847035.

### 2.8 Reverse transcription quantitative PCR (qRT-PCR)

The samples were collected and frozen using liquid nitrogen. Total RNA was extracted from mycelia using the TrIzol REAgent (Biomed, Beijing, China) according to the manufacturer’s instructions. Reverse transcription of cDNA was performed by reverse transcription kits according to the manufacturer’s instructions (Thermo Fisher Scientific, MA). The qRT-PCR assays were performed on seven genes detected as highly differentially expressed *via* RNA-seq. The seven selected genes are Atg3 (which can catalyze conserved reactions in glycolysis) (Graham et al., 2002), CLAH10 (a stress response enzymes) (Mukherjee et al., 2019), nuo-51 (a ubiquinone oxidoreductase) (Ryčovská et al., 2000), SDH2 (a component of the tricarboxylic acid cycle) (Huang and Millar, 2013), msh-2 (which is necessary for microsatellite stability and maintenance of genome integrity) (Kunkel and Erie, 2015) ATPeV1A (an ATP-driven enzyme) (Beyenbach and Wieczorek., 2006). gpi17 (a subunit of GPI-Transamidase) (Gamage et al., 2017). The reaction system of qRT-PCR included 5 μl SYBR Green Mix, 3 μl RNA-free water, 1 μl a pair of specific primers (0.5 μl forward primer and 0.5 μl reverse primer) and 1 μl cDNA templates. All the sequences of forward and reverse primers are shown in Table 1. The qRT-PCR procedure was set as follows: 95°C for 5 min, followed 40 cycles of 95°C for 10 s and 60°C for 30 s, with a final extension of 72°C for 60 s. According to the fluorescence threshold results (Ct values), all data were normalized to the housekeeping gene Actin (Shen et al., 2020) as the internal reference gene, and expression levels were calculated using the ΔΔCt method, as previously described (Schmittgen and Livak, 2001; Chen et al., 2020).

**TABLE 1:** The primers used for qRT-PCR.

Gene	Sequences (5′-3′)
At3g	Forward:CATCTCTCAACGCTCACC
	Reverse: GCA​TAT​GTC​AAA​CGC​TCA​GG
CHLA10	Forward: TCA​GGT​CTT​CCG​TCA​GCA​A
	Reverse: CAGCAACAACGGCACCA
SDH2	Forward: CATCAAGCCCTACCTCC
	Reverse: TTCCACCAGTAAGACGG
rnp-8	Forward: ATCTCCCTCTACACGCTT
	Reverse: AACCGGCAGAACTAGACA
nuo-51	Forward: GAT​CAT​GCG​AAA​GGA​CCC​ACA
	Reverse: CTC​CTC​GAC​GAA​TTC​ACC​T
ATPeV1A	Forward: GAACATCGCCAAGTCCA
	Reverse:GTACCCCAGACATCACC
gpi17	Forward: TACTTCCCAGACGAGCAC
	Reverse:CTTCTTCTCGCCTGCATC
Actin	Forward: CAC​CAC​CTT​CAA​CTC​CAT​CA
	Reverse: TCG​GAG​AGA​CCA​GGG​TAC​AT

### 2.9 Statistics

The datas were analyzed using parametric statistical tests. Three independent biological replicates were performed for each assay. One-way analysis of variance (ANOVA) was used to analyze differences between the 1,8-cineole and control group. Statistical Product and Service Solutions (SPSS) 18.0 software (SPSS Inc., Chicago, IL, United States) was used for statistical analysis. *p* < 0.05 was considered statistically significant.

## 3 Results

### 3.1 1,8-cineole inhibits *F. solani* species complex biofilm

**FIGURE 1:**
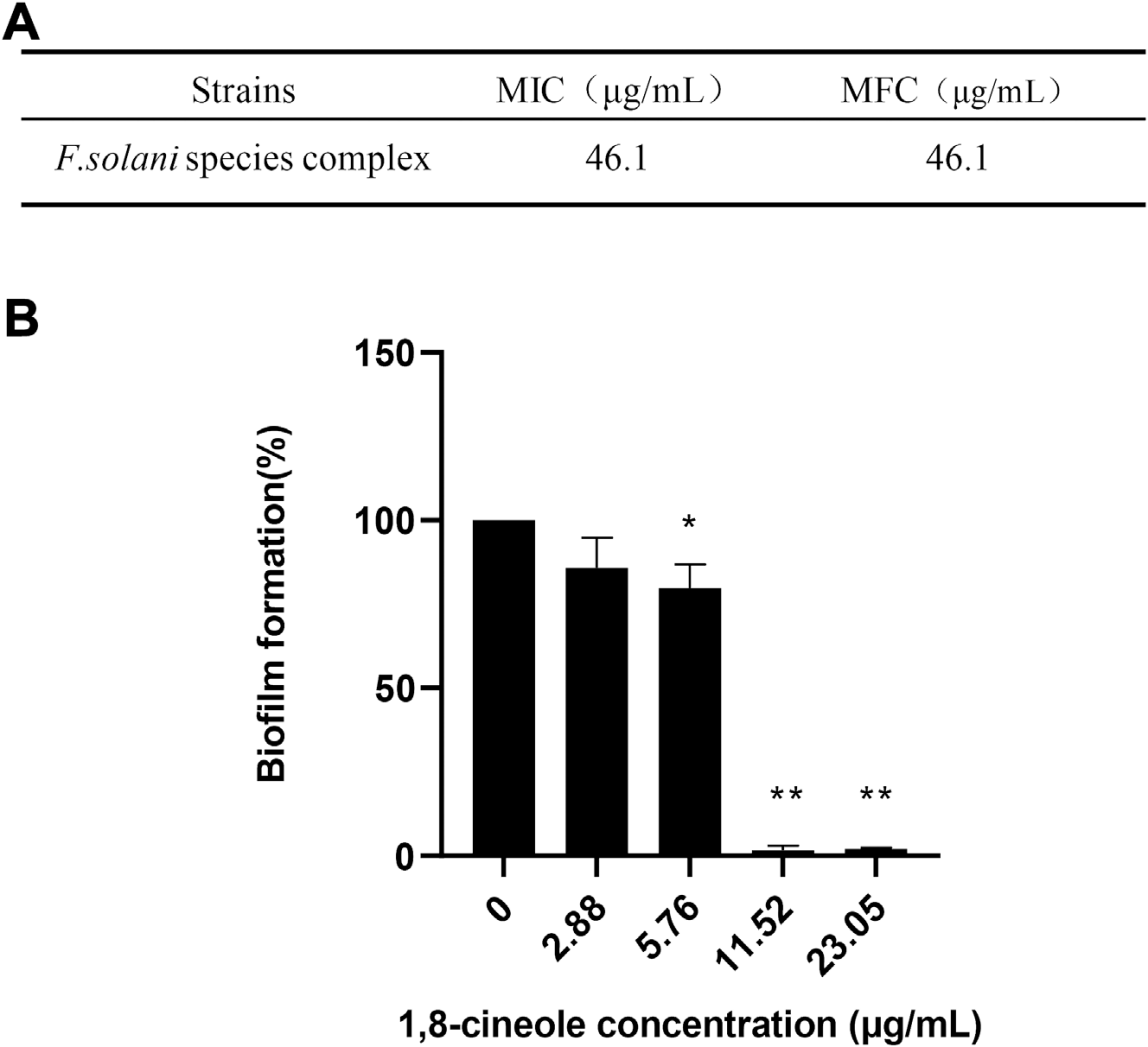
1,8-cineole inhibits F. solani species complex biofilm formation *in vitro*. Biofilm formation was evaluated using an XTT reduction assay, and the results are presented as the percent of 1,8-cineole treated biofilm relative to control biofilm formed without treatment. **(A)** The MICs and MFCs of 1,8-cineole against *F. solani* species complex (46.1–2.88 μg/ml). **(B)** Effects of different concentrations of 1,8-cineole on biofilm formation. The results represent means ± standard deviations for three independent experiments. *, *p* < 0.05; **, *p* < 0.01.

In this study, we firstly evaluated the antifungal effect of 1,8-cineole against *F. solani* species complex. Antifungal susceptibility testing results showed that the 1,8-cineole has a same MIC and MFC against *F. solani* species complex at 46.1 μg/ml (Figure 1A). To determine the inhibitory effect of 1,8-cineole against *F. solani* species complex biofilm, a XTT reduction assay was performed. The results showed that 1,8-cineole has a good anti-biofilm activity against *F. solani* species complex biofilm in a dose-dependent-manner (Figure 1B). Notably, 1,8-cineole inhibited 98.24% (*p* < 0.01) biofilm formation at a concentration of 11.52 μg/ml.

### 3.2 1,8-cineole inhibits the formation of *F. solani* species complex biofilm structure

**FIGURE 2:**
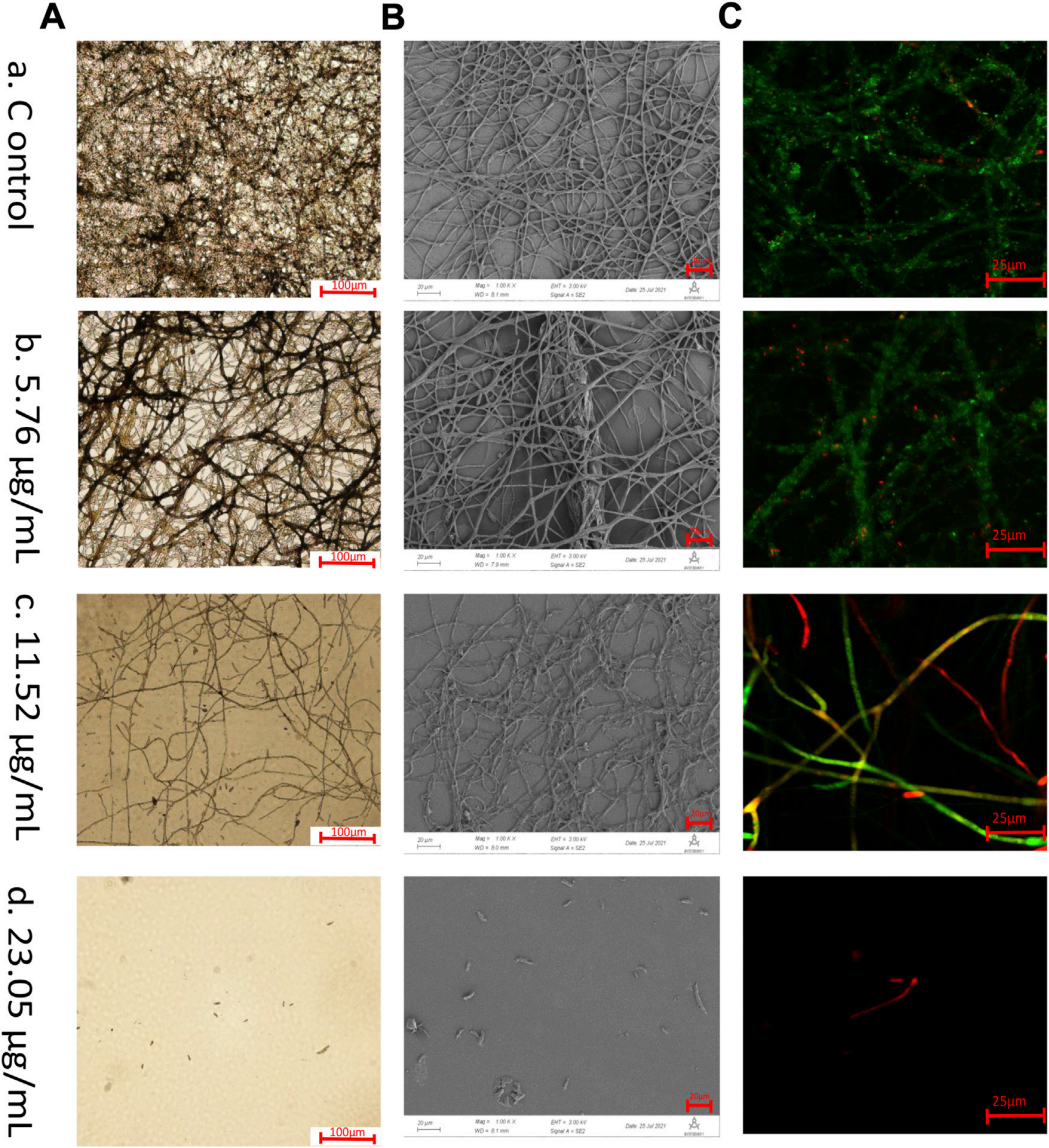
Microscopic observation. Biofilms of *F. solani* species complex are visualized by different microscopic techniques. The *F. solani* species complex cells were transferred to RPMI 1640 media with different concentrations of 1,8-cineole at 27°C for 72 h. The samples were treated at **(A)** 5.76 μg/ml, **(B)** 11.52 μg/ml, **(C)** 23.05 μg/ml, and **(D)** 46.1 μg/ml of 1,8-cineole, respectively.**(A)** Optical microscopic images of biofilm formation under 1,8-cineole treatment. Scale bar is 100 μm (100x). **(B)** Scanning electron microscopy images of biofilm formation under 1,8-cineole treatment. Scale bar is 20 μm (1,000x). The arrow indicated that the biofilm has multilayer mycelium structure. **(C)** CLSM images of biofilm formation under 1,8-cineole treatment. NucGreen is green nucleic acid staining and EthD-III is red nucleic acid staining. Red represents dead cells, green represents living cells, and yellow represents weakened cells Scale bar is 25 μm (200x).

Optical microscope, SEM, CLSM techniques were used to observe *F. solani* species complex biofilms treated with 23.05–5.76 μg/ml 1,8-cineole as well as untreated biofilms (Figure 2). Optical microscope, with low magnifications (Figure 2A), provided an overview and preliminary inspection of *F. solani* species complex biofilms were affected by different concentrations of 1,8-cineole treatment, and showed untreated *F. solani* species complex biofilm with dense fungal growth (Figure 2Aa). The biofilm gradually decreased after treating with 1,8-cineole. Notably, 23.05 μg/ml 1,8-cineole could completely inhibit the growth of biofilm (Figure 2Ab, Ac, Ad).

SEM with a higher magnification and larger depth of focus, which can provide three-dimensional close up inspection of biofilms, allowing a better assessment of the inhibition of *F. solani* species complex biofilms formation that treated with 1,8-cineole (Figure 2B). After 72 h incubation, the untreated *F. solani* species complex formed a mature biofilm, fusion of hyphae, interconnection or anastomosis with well-development channels between several layers of hyphae and production of ECM (Figure 2Ba). After 11.52 μg/ml 1,8-cineole treatment, the morphological arrangement of the hyphae was significantly different from that of the control group, with significantly reduced ECM, damaged hyphae with flattened structures, and reduced hyphal density (Figure 2Bc).

To evaluate the 1,8-cineole killing efficiency against *F. solani* species complex biofilm cells during biofilm formation, NucGreen - EthD-III dual staining was performed and observed by CLSM (Figure 2C). In the growth control group, it was observed that the cells grew well, with green fluorescence. The number of cell death gradually increased with the increase of 1,8-cineole concentration (Figure 2Ca, Cb, Cc), with an increasing red fluorescent signal. At 23.05 μg/ml of 1,8-cineole, there was no cell survived (Figure 2Cd).

The hyphae in *F. solani* species complex biofilms were inhibited by 1,8-cineole in a dose-dependent manner, and more specifically, after 23.05 μg/ml of 1,8-cineole treatment, *F. solani* species complex cells maintained the state of spore and criss-crossing true hyphae could not be observed (Figure 2Ad, 2Bd, 2Cd).

### 3.3 1,8-cineole inhibits adhesion of *F. solani* species complex

**FIGURE 3:**
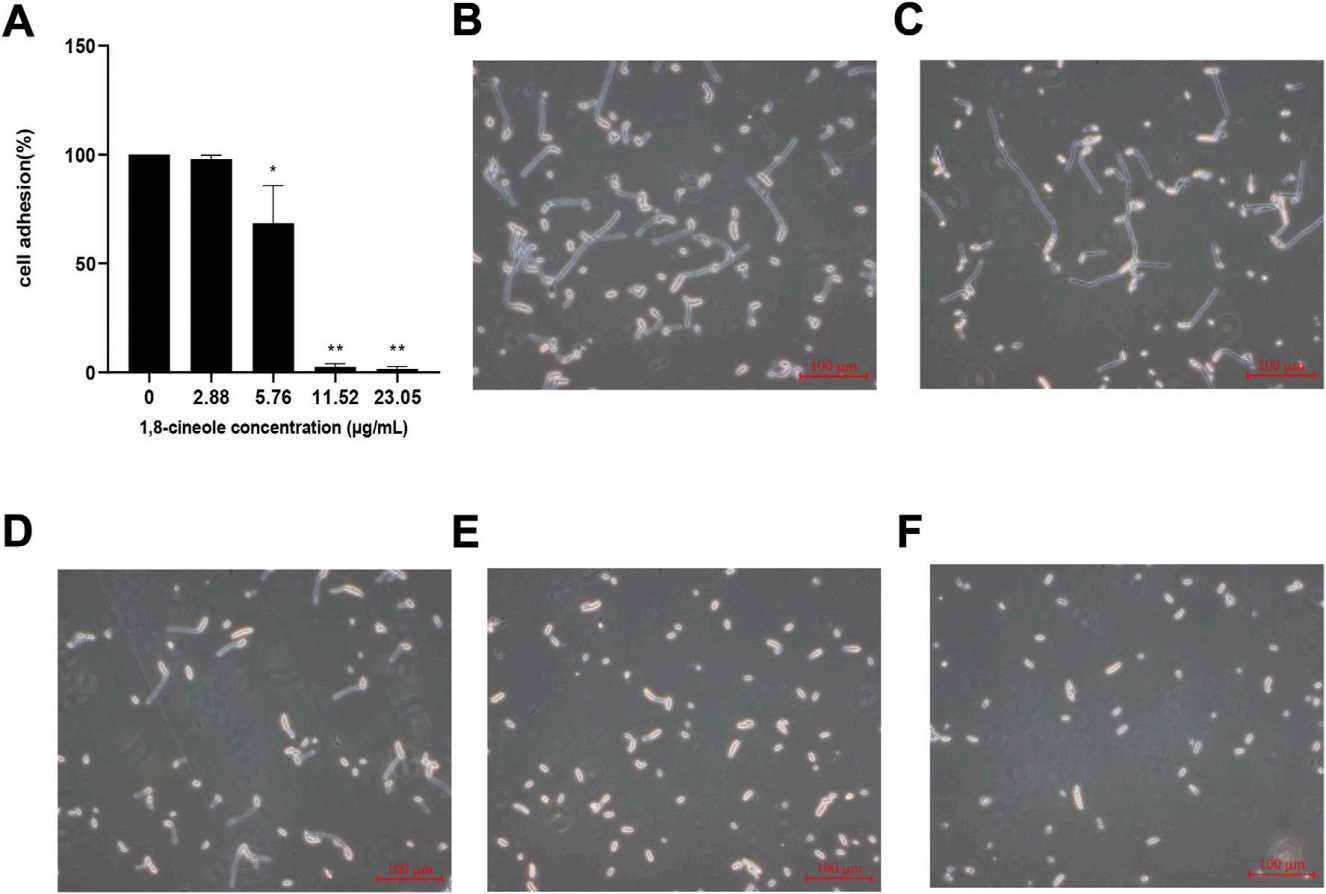
Effect of different concentrations of 1,8-cineole on adhesion and hypha formation. **(A)** Effects of different concentrations of 1,8-cineole on adhesion. Exponentially growing *F. solani* species complex cells were transferred to RPMI 1640 media with different concentrations of 1,8-cineole at 27°C for 4 h. In adhesion assay, exponentially growing *F. solani* species complex cells were incubated with different concentrations of 1,8-cineole for 4 h and was evaluated using an XTT reduction assay. (C ∼ F) Effects of different concentrations of 1,8-cineole on hypha formation. Exponentially growing *F. solani* species complex cells were transferred to RPMI 1640 media with different concentrations of 1,8-cineole at 27°C for 6 h. The group of control **(B)** and the concentration of 2.88 μg/ml **(C)**, 5.76 μg/ml **(D)**, 11.52 μg/ml **(E)**, and 23.05 μg/ml **(F)** 1,8-cineole, respectively. The results represent means ± standard deviations for three independent experiments. *, *p* < 0.05; **, *p* < 0.01.

A cell adhesion assay was performed to determine the effect of 1,8-cineole on attachment of *F. solani* species complex strain (Figure 3A). Compared with the control cells, a significant reduction (*p* < 0.05) in the adhesion of cells to the plastic surfaces was observed under the treatment of 1,8-cineole. The results showed that *F. solani* species complex adhesion was reduced 98% at the 11.52 μg/ml concentration of 1,8-cineole treatment (*p* < 0.01).

### 3.4 1,8-cineole inhibits hypha formation of *F. solani* species complex

The effect of 1,8-cineole on *F. solani* species complex hypha formation was further evaluated by incubating *F. solani* species complex in RPMI 1640 media for 72 h (Figure 3B∼ 3F). 1,8-cineole inhibited hypha formation in a dose-dependent manner. After treatment with 1,8-cineole at a concentration of 5.76 μg/ml, the hypha of the *F. solani* species complex began to be shorten. Interestingly, hypha formation was completely inhibited by 23.05 μg/ml of 1,8-cineole in RPMI 1640 medium and all *F. solani* species complex cells were maintained as spores.

### 3.5 1,8-cineole decreases the formation of extracellular matrix of *F. solani* species complex biofilm

**FIGURE 4:**
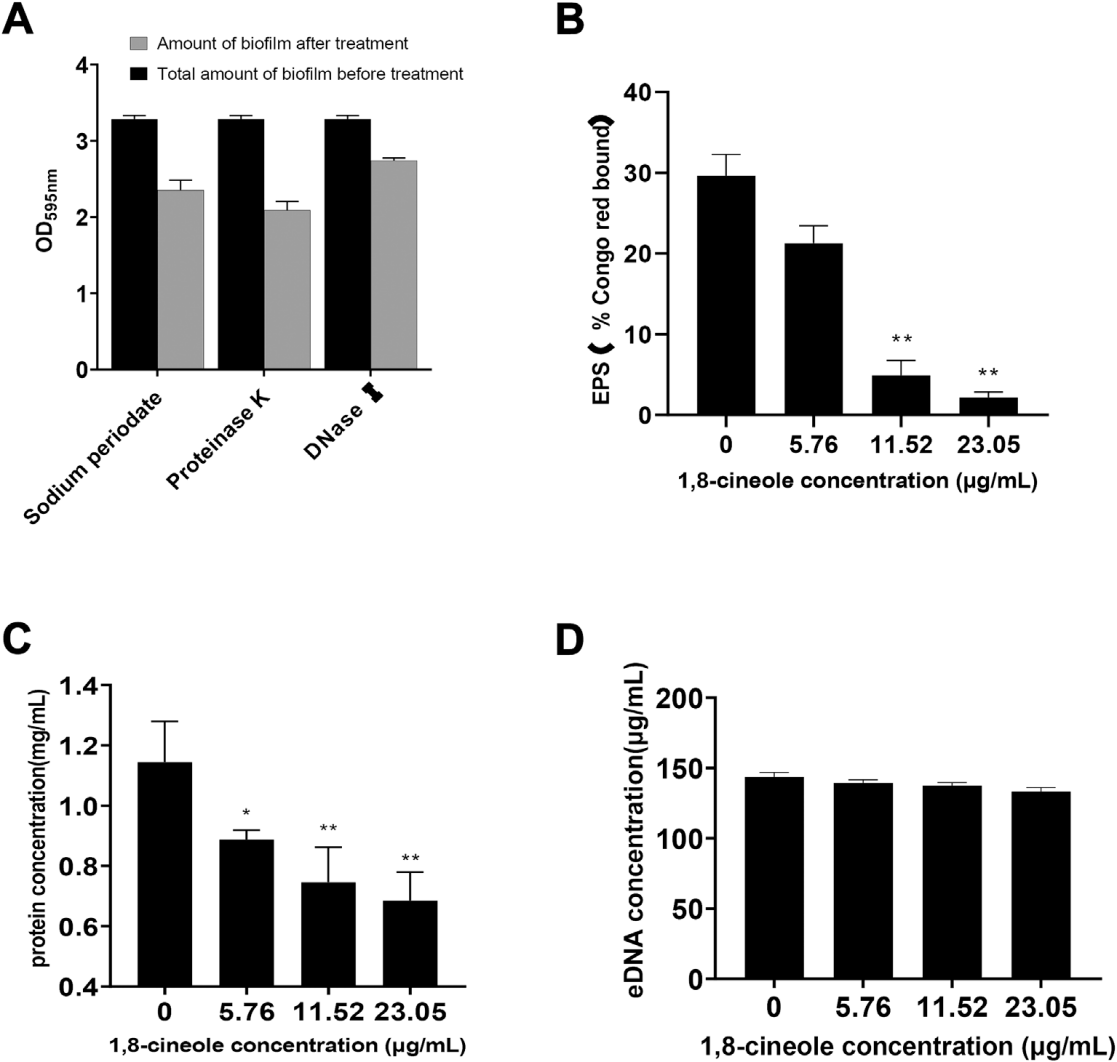
Effect of different concentrations of 1,8-cineole on Extracellular matrix. **(A)** The major components of *F. solani* species complex extracellular matrix were analyzed by adding to the preformed biofilm protease K to degrade proteins, DNase I to degrade DNA and sodium periodate to degrade β-1,6-linked polysaccharides. Biofilms were developed in 24-well plates incubated at 27 C for 72 h in RPMI 1640 medium and quantified by crystal violet assay. **(B)** The effect of 1,8-cineole on the extracellular polysaccharide (EPS) was evaluated using Congo red test. **(C)** Effect of 1,8-cineole on extracellular proteins was determined by Coomassie Brilliant Blue staining method. **(D)** The impact of various concentrations of 1,8-cineole on eDNA was evaluated by using Phenol chloroform method. Comparisons between absorbance levels revealed significant differences. *, *p* < 0.05; **, *p* < 0.01.

To corroborate the molecules involved in the formation of biofilm of *F. solani* species complex, it was subjected to different treatments by detachment assay: protein degradation, polysaccharide degradation and DNA degradation. The biofilm was quantified by CV assay, in which the biomass formed by *F. solani* species complex was reduced by 28.44% with sodium periodate, 36.38% with Proteinase K, and 16.56% with DNaseⅠ (Figure 4A).

Compared with the control group, the extracellular polysaccharide and protein formation was reduced in 1,8-cineole treatment group (Figures 4B,C). When the concentration of 1,8-cineole reached to 11.52 μg/ml, extracellular polysaccharide and protein content were decreased by 83.36% and 35.01%, respectively. However, no significant differences in the leakage of eDNA were observed for control group and 1,8-cineole treated groups, at the experimental dose (Figure 4D).

### 3.6 1,8-cineole inhibited biofilms formation exhibit a novel transcriptome

#### 3.6.1 Illuminate sequencing and *de novo* transcriptome assembly

In order to avoid the influence of cell growth inhibition on the expression of mRNA, 1,8-cineole at 5.76 μg/ml was chosen for RNA-seq analysis. *F. solani* species complex biofilm with 5.76 μg/ml 1,8-cineole treatment was named “treatment group”, and *F. solani* species complex biofilm without 1,8-cineole treatment was named “control group”.

**FIGURE 5:**
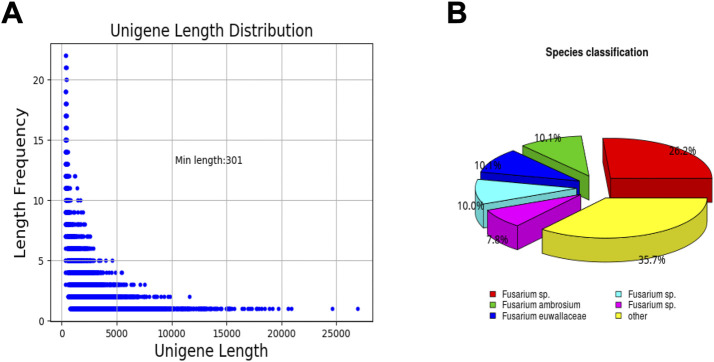
Unigene length distribution and species classification. **(A)** unigene length distribution. **(B)** species classification.

To obtain a comprehensive overview of *F. solani* species complex biofilm transcriptome, a cDNA library from *F. solani* species complex biofilm was constructed and sequenced using Illumina RNA-seq. A total of 23233245 and 21189058 reads were generated from the control and treatment group, respectively (Table 2). The average length of raw reads was 3,028 nucleotides (Table 3). After removing of adapter sequences, ambiguous reads and low quality reads, 22161005 and 19733412 high-quality clean reads were obtained with an average GC content of 56.16% and 56.74% (Table 2). Finally, all short sequences were assembled into 29766246 unigenes with an average length of 2,185 bp, a maximum length of 26,965 bp and an N50 of 3,524 bp. The length distribution of the unigenes was illustrated in Figure 5A. The sequences ranging from 300 bp to 1,000 bp in length accounted for nearly 36.99% of the total. Up to 3,261 unigenes (23.94%) and 5,322 unigenes (39.07%) were 1,000 bp to 2000 bp and >2000 bp in length, respectively (Table 4).

**TABLE 2:** *De novo* transcriptome analysis in *F. solani* species complex biofilm and identification of critical genes involved in 1,8-cineole treatment.

Samples	Raw reads	Clean reads	Q20	GC (%)
Control	23233245	22161005	98.18	56.16
Treatment	21189058	19733412	98.29	56.74

**TABLE 3:** Summary statistic of *F. solani* species complex biofilm transcriptome assembly.

Type	Min length	Mean length	Median length	Max length	N50	Total nucleotides
Transcript	301	3,028	2,120	26,965	4,963	77261407
Unigene	301	2,185	1,492	26,965	3,524	29766246

**TABLE 4:** Length distribution of *F. solani* species complex biofilm unigenes.

Transcript length interval	300-500 bp	500-1 kbp	1k-2 kbp	>2 kbp	Total
Number of Unigenes	2,400	2,638	3,261	5,322	13,621

#### 3.6.2 Transcriptome annotation and functional classification

After eliminating low-quality and short-length sequences, 13,621 unigenes were subjected to annotation analysis by matching sequences against Nr, Pfam and Swiss-prot databases. 11,190 unigenes (82.15% of the total) can be matched in Nr database, 8,276 unigenes (60.75% of the total) matched Pfam, and 7,200 unigenes (52.85% of the total) matched in Swiss-prot (Table 5). By comparing and annotating with the Nr database, the similarity between the gene sequence of this species with its relatives and the functional information can be obtained. The results showed that the gene sequences covered 44% of the *Fusarium* species. In addition, 10.1% and 10.1% of the gene sequences were matched to *Fusarium ambrosium* and *Fusarium euwallaceae*, respectively (Figure 5B).

**TABLE 5:** Summary statistic of *F. solani* species complex biofilm transcriptome annotation.

	Number of unigenes	Percentage (%)
Annotated in NR	11,190	82.15
Annotated in SwissProt	7,200	52.85
Annotated in PFAM	8,276	60.75
Total Unigenes	13,621	100

**FIGURE 6:**
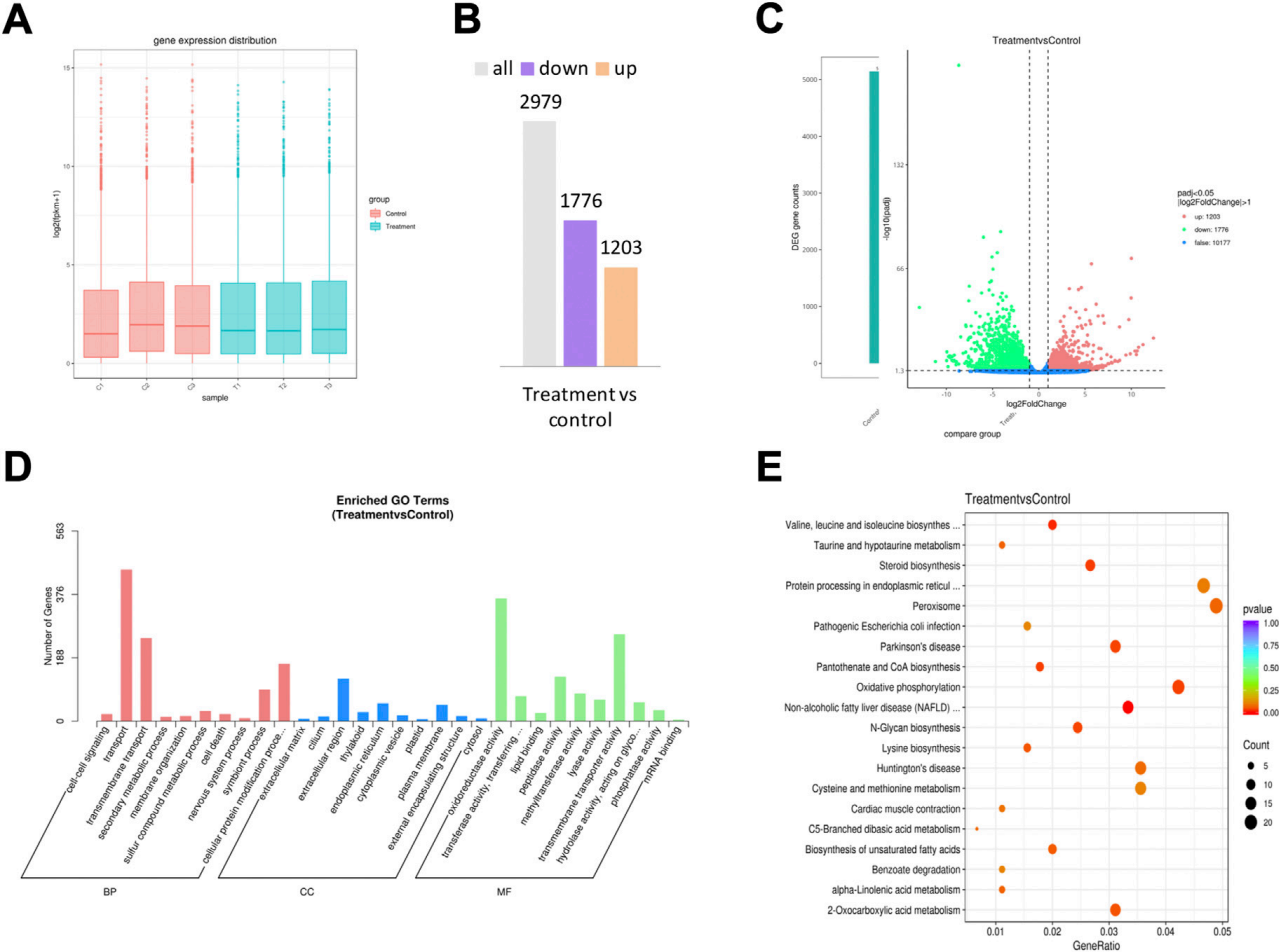
RNA sequencing analysis. *F. solani* species complex cells under treatment with 5.76 μg/ml of 1,8-cineole at 27 C for 72 h. **(A)** The box plot shows the variation in mRNA expression. **(B)** Statistical chart of DEGs. **(C)** Volcano plots of DEGs for *F. solani* species complex biofilm formed in response to 5.76 μg/ml 1,8-cineole; the red dots represent the up-regulated genes, the green dots represent the down-regulated genes, and the blue dotted line represents the threshold line of differential gene screening criteria. **(D)** GO analysis and **(E)** KEGG pathway classification analysis of upregulated and downregulated genes.

Significant transcriptional changes were observed in the *F. solani* species complex biofilms after a 72 h treatment with 1,8-cineole (Figure 6). The raw expression levels of each sample were shown in the boxplots, and little variability was observed between the sample (Figure 6A). A total of 2,979 differentially expressed genes (DEGs) were seen in the 1,8-cineole-treated biofilms group, compared to the control group (Figure 6B), of which 1,203 were upregulated and 1776 downregulated (Figure 6C) (log_2_ fold change ≥1, *p* < 0.05).

GO terms were assigned to assemble unigenes and provided defined ontologies to express gene product properties. GO function classification analysis was performed to identify alterations in the biological processes (BP), cellular composition (CC), and molecular functions (MF). GO revealed that the DEGs were mainly related to transport, transmembrane transport, extracellular region, oxidoreductase activity and transmembrane transporter activity (Figure 6D).

KEGG analysis demonstrated the biological pathways in which the unigenes are involved. Assembled unigenes were compared with the KEGG database using BLASTx and the corresponding pathways were established. KEGG pathway classification analysis results showed that the DEGs were mainly enriched in the pathways of valine, leucine and isoleucine biosynthesis, pantothenate and COA biosynthesis, 2- oxocarboxylic acid metabolism and oxidative phosphorylation (Figure 6E).

### 3.7 Validation of RNA-Sequencing data using qRT-PCR

**FIGURE 7:**
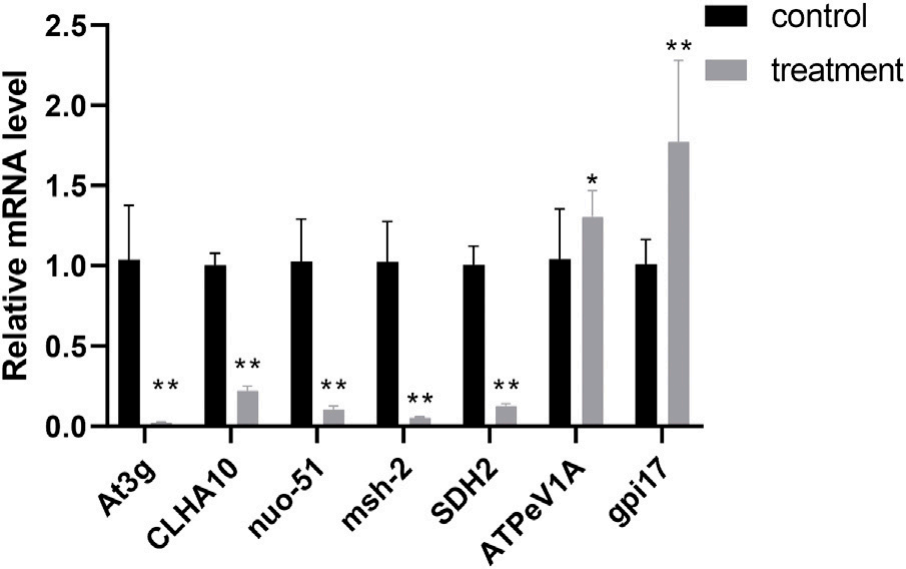
Correlation of expression level analyzed by qRT-PCR with data obtained using RNA-Seq platform. The data represent mean ± SD (n = 4). *, *p* < 0.05; **, *p* < 0.01.

To validate the DEGs expression levels of *F. solani* species complex that identified by RNA-Seq analysis, 7 genes (5 up-regulated and 2 down-regulated) with highest fold changes were selected from stages of biofilm matura and qRT-PCR was performed. The results of qRT-PCR confirmed the gene expression trend observed in RNA-seq data (Figure 7).

## 4 Discussion

1,8-cineole has a good inhibitory effect on many fungi, such as *Fusarium oxysporum*, *Fusarium sporotrichioides*, *Aspergillus tubingensis* (Morcia et al., 2012). In this study, we demonstrated that 1,8-cineole can effectively inhibit *F. solani* species complex biofilm formation *in vitro*. To get more deep insight in the inhibition biofilm formation activity of 1,8-cineole against *F. solani* species complex, different microscopy assays were performed. The results of silver staining and SEM showed that the biofilm and ECM decreased with 1,8-cineole treatment. We observed that most of the ECM wrapped the mycelium, causing adhesion between the mycelium, while a small part of the extracellular matrix was “blanket” shaped, covering the mycelium (as shown in Figure 2B). This morphology of ECM was observed in this experiment has a similar structure with Chen. B (Chen et al., 2019). The formation of ECM will greatly increase the drug resistance of fungus in the biofilm state. Noteworthy, a large quantity of well-organized hyphae can be seen, which was consistent with images illustrating the biofilm of *F. oxysporum* (Veiga et al., 2021).

Since 1,8-cineole has a good inhibitory effect on the formation of biofilm, we further investigated the effect of 1,8-cineole on the formation stage of biofilm. The cycle of biofilm is adhesion, hyphal formation, maturation and dispersion (Pereira et al., 2021). Among them, adhesion and hyphal formation are key factors for biofilm formation and maturation. Adhesion is the first step for biofilm formation, which makes planktonic cells adhere to form colonies. The spores adhered to the surfaces, continuously grow and multiplying, and then further forming hyphae. The transformation from spores to hyphal phases at this stage is considered to be the main pathogenic factor (Huang et al., 2020). Our results demonstrated that 5.76 μg/ml 1,8-cineole could effectively reduce the adhesion of *F. solani* species complex and the formation of hypha (*p* < 0.05). These results indicated that the effect of 1,8-cineole on the biofilm may be attributed to inhibiting adhesion and hypha formation.

In addition, ECM is produced during the formation of biofilm, which is a complex mixture of macromolecules including proteins, exopolysaccharides and eDNA. Previous studies have shown that the extracellular matrix (ECM) formed by biofilm is the direct reason of fungal resistance to commonly used antifungal drugs (Gupta et al., 2016; Nogueira Brilhante et al., 2017). Saima Muzammil found that the content of protein, extracellular polysaccharide and eDNA in extracellular matrix of *Acinetobacter baumannii* were reduced after Aluminium oxide nanoparticles treatment (Muzammil et al., 2020). Therefore, we hypothesised that 1,8-cineole could affect the formation of biofilm by affecting the composition of ECM. Interestingly, the results showed that 1,8-cineole could inhibit extracellular protein and extracellular polysaccharide.

To investigate the effect of 1,8-cineole on gene expression of *F. solani* biofilm, transcriptomics was then used. KEGG and GO enrichment analyses were performed to determine the effect of biofilm formation while 1,8-cineole was added. There were different changes of several pathways associated with substance and energy metabolism and genetic information.

In view of the lipophilicity of essential oil, microbial cell plasma membrane is considered as the target of essential oil or its volatile components (Wang et al., 2019; Nunes et al., 2021). Essential oils can affect the expression of genes involved in cell membrane related pathways, such as fatty acid biosynthesis (FAS2) and sterol biosynthesis (ERG), thereby damaging cell membrane integrity (OuYang et al., 2016). ERG genes were upregulated in *C. albicans* after exposure to the itraconazole. (De Backer et al., 2001). Our results showed that genes related to steroid biosynthesis, such as sterol 14 α-demethylase (CYP51), sterol synthase (ERG7) and sterol reductase (ERG4) were up-regulated in *F. solani*. Genes involved in phosphatidylic acid biosynthesis were down regulated 6.2 times after treatment with 1,8-cineole, and phosphatidylserine decarboxylase (PSD) was down regulated 2.7 times. However, diacylglycerol kinase (DGK1) and choline kinase (CKI1) were up-regulated. Based on this phenomenon, the results indicated 1,8-cineole may affect membrane fluidity by changing the expression of membrane component related genes. In addition, the results indicated 1,8-cineole may penetrate the cell membrane and destroy the organelles without damaging the membrane.

Energy and substance metabolisms are the basis of life activity, especially under stress conditions. *M. alternifolia* essential oil can affect the respiration and metabolism of *Sitophilus zeamais* (Liao et al., 2016). This study found that almost all of differential genes encoding carbohydrate metabolism, amino acid metabolism and fatty acid metabolism were down regulated after 72 h of treatment with 1,8-cineole (Supplementary Table S1). Mitochondria are one of the important potential targets of essential oils (Wu et al., 2009). Turmeric EO inhibited the activities of ergosterol synthesis, mitochondrial ATPase, succinate dehydrogenase, and malate dehydrogenase (Hu et al., 2017). Transcriptome sequencing results showed that the genes encoding glycolysis related enzymes, including PGK, GPI, eno, and TPI were down regulated. Moreover, the genes encoding the TCA cycle including malate dehydrogenase (mdh2) and aconitase (ACO) were inhibited. Glucose is metabolized through TCA cycle to produce ATP and reduce NADH (Fernie et al., 2004). The glycolytic pathway and oxidative phosphorylation (Song et al., 2019) create the majority of the ATP necessary for fungal growth, reproduction and infection. Oxidative phosphorylation in mitochondria is the main source of energy required for life activities. ATP synthesized in this way accounts for about 95% of the total (Chinopoulos and Seyfried, 2018). Electrons produce a proton gradient across the membrane through the electron transport chain and produce ATP to provide energy for cells. Ruopeng Yang made cinnamaldehyde, eugenol or carvacrol into a compound nanoemulsion, which exerts antifungal activity by influencing the cellular respiration and proton transmembrane biological processes (Yang et al., 2021). Our data showed that the oxidative phosphorylation pathway was inhibited, in which NADH dehydrogenase (ubiquinone) (Complex I), succinate dehydrogenase (SDHB) (Complex II), cytochrome c oxidase (cox1) (Complex III), Fe-S protein 1 (NDUFS1) and other enzymes were down regulated. The results indicated 1,8-cineole caused the transmembrane transport of protons and electrons in mitochondria affects the production of ATP and then made *F. solani* starved of energy and dead. Meanwhile, the decline of the complex I, II, III and IV activity also led to the destruction of mitochondria.

Essential oil treatment can affect genes related to genetic information processing (transcription, translation, replication and repair (Lv et al., 2018). Interestingly, in this study, transcriptional regulatory-related genes (RPAC2 and RPC3) were downregulated in biofilm cells exposed to 1,8-cineole (Supplementary Table S2). Therefore, the results indicated that 1,8-cineole may interfere with the regulatory network during biofilm formation. Besides, the downregulated enolase gene with 1,8-cineole treatment suggested fungal adhesion was inhibited. Moreover, 1,8-cineole had reduced the activity of proteinase (glnS, asps and cycS) which made the formation of ECM of the *F. solani* biofilm after 1,8-cineole treatment was blocked. This means that the anti-biofilm formation ability of 1,8-cineole could not only interfere with cellular pathway, but also target the ECM of biofilm.

Collectively, the probable anti-biofilm mechanism of 1,8-cineole against *F. solani* biofilm formation involves multiple pathways and components. In the process of biofilm formation, 1,8-cineole first interacts with different biomolecules in ECM and inactivates various enzymes, which inhibits the formation of ECM. And then 1,8-cineole pass through ECM. After reaching the cell surface, 1,8-cineole penetrated the cell through the membrane. It can inhibit ergosterol synthesis in the cell membrane and change the permeability of the cell membrane. In the cytoplasm, 1,8-cineole mediates antifungal activity by attacking multiple pathways at the same time. Firstly, 1,8-cineole interferes with the metabolism of ability, including oxidative phosphorylation, glycolysis/gluconeogenesis and other energy metabolism pathways. In addtition, it can affect the production of ATP by changing the activity of mitochondrial complex I, II, III and IV; secondly, 1,8-cineole interferes with the transcriptional expression of biosynthetic pathway genes and inhibits the activity of protease.

In conclusion, the formation of *F. solani* species complex biofilm was greatly inhibited by 1,8-cineole. Further, the findings in our study suggested 1,8-cineole downregulated of ergosterol biosynthetic genes, inhibited adhesion, hindered the synthesis of ECM and interfered mitochondrial activity. This further demonstrated the value of 1,8-cineole as an anti-biofilm formation agent against *F. solani*. To our best knowledge, this research is the first study to report the phenotype and expression profiles of *F. solani* in biofilms exposed to 1,8-cineole.

## Data Availability

The datasets presented in this study can be found in online repositories. The names of the repository/repositories and accession number(s) can be found below: https://www.ncbi.nlm.nih.gov/, PRJNA847035.
