# Supplementary material for: Study on the anti-biofilm mechanism of 1,8-cineole against Fusarium solani species complex
Source: Front Pharmacol. 2022 Oct 14;13:1010593. doi: 10.3389/fphar.2022.1010593 (PMC9624185; doi:10.3389/fphar.2022.1010593)
Supplement: Supplementary file 1 [file DataSheet1.docx]

Supplementary table 1 summary of a part of major genes related to energy and substance metabolism affected in *F. solani* exposed to 1,8-cineole

| ID | KEGG pathway | Enzyme | Fold change  (T/C) |
| --- | --- | --- | --- |
| Oxidative phosphorylation | | | |
| Cluster-2231.4464 | K03934 | Ndufs1;NADH dehydrogenase (ubiquinone) Fe-S protein 1 [EC:[7.1.1.2](https://www.kegg.jp/entry/7.1.1.2)] | -2.254 |
| Cluster-2231.4052 | K03935 | Ndufs2;NADH dehydrogenase (ubiquinone) Fe-S protein 2 [EC:[7.1.1.2](https://www.kegg.jp/entry/7.1.1.2)] | -1.8843 |
| Cluster-2231.2968 | K03939 | Ndufs6;NADH dehydrogenase (ubiquinone) Fe-S protein 6[EC:[7.1.1.2](https://www.kegg.jp/entry/7.1.1.2)] | -1.8949 |
| Cluster-2231.4749 | K03942 | Ndufv1;NADH dehydrogenase (ubiquinone) flavoprotein 1 [EC:[7.1.1.2](https://www.kegg.jp/entry/7.1.1.2)] | -2.6116 |
| Cluster-2231.5117 | K03952 | Ndufa8;NADH dehydrogenase (ubiquinone) 1 alpha subcomplex subunit 8 | -1.963 |
| Cluster-2231.4743 | K03953 | Ndufa9;NADH dehydrogenase (ubiquinone) 1 alpha subcomplex subunit 9 | -2.4132 |
| Cluster-2231.4720 | K03964 | Ndufb8;NADH dehydrogenase (ubiquinone) 1 beta subcomplex subunit 8 | -2.2295 |
| Cluster-2231.3853 | K00235 | SDHB;succinate dehydrogenase (ubiquinone) iron-sulfur subunit [EC:[1.3.5.1](https://www.kegg.jp/entry/1.3.5.1)] | -3.77 |
| Cluster-2231.4527 | K00413 | Cyt1; ubiquinol-cytochrome c reductase cytochrome b/c1 subunit | -2.7975 |
| Cluster-2231.4652 | K00415 | QCR2;ubiquinol-cytochrome c reductase core subunit 2 | -2.3898 |
| Cluster-2414.0 | K02256 | COX1; cytochrome c oxidase subunit 1 [EC:[7.1.1.9](https://www.kegg.jp/entry/7.1.1.9)] | -2.3898 |
| Cluster-2231.4262 | K02264 | COX5A;cytochrome c oxidase subunit 5a | -2.8683 |
| Cluster-2231.3908 | K02266 | COX6A;cytochrome c oxidase subunit 6a | -2.0398 |
| Citrate cycle (TCA cycle) | | | |
| Cluster-2231.6458 | K00026 | MDH2; malate dehydrogenase [EC:[1.1.1.37](https://www.kegg.jp/entry/1.1.1.37)] | -2.1346 |
| Cluster-2231.3853 | K00235 | SDHB; succinate dehydrogenase (ubiquinone) flavoprotein subunit [EC:[1.3.5.1](https://www.kegg.jp/entry/1.3.5.1)] | -3.77 |
| Cluster-2231.2470 | K01681 | ACO; aconitate hydratase [EC:[4.2.1.3](https://www.kegg.jp/entry/4.2.1.3)] | -6.8091 |
| Glycolysis / Gluconeogenesis | | | |
| Cluster-2231.4216 | K01810 | GPI, pgi; glucose-6-phosphate isomerase [EC:[5.3.1.9](https://www.kegg.jp/entry/5.3.1.9)] | 1.7154 |
| Cluster-2231.3506 | K01803 | TPI, tpiA; triosephosphate isomerase (TIM) [EC:[5.3.1.1](https://www.kegg.jp/entry/5.3.1.1)] | -1.2924 |
| Cluster-2231.4426 | K00927 | PGK, pgk; phosphoglycerate kinase [EC:[2.7.2.3](https://www.kegg.jp/entry/2.7.2.3)] | -1.3094 |
| Cluster-2231.4099 | K15633 | gpmI; 2,3-bisphosphoglycerate-independent phosphoglycerate mutase [EC:[5.4.2.12](https://www.kegg.jp/entry/5.4.2.12)] | -2.5931 |
| Cluster-2231.6211 | K01689 | ENO, eno; enolase [EC:[4.2.1.11](https://www.kegg.jp/entry/4.2.1.11)] | -6.2432 |
| Cluster-2231.4222 | K01568 | PDC, pdc; pyruvate decarboxylase [EC:[4.1.1.1](https://www.kegg.jp/entry/4.1.1.1)] | -1.8707 |
| Cluster-2231.5384 | K00016 | LDH, ldh; L-lactate dehydrogenase [EC:[1.1.1.27](https://www.kegg.jp/entry/1.1.1.27)] | -7.4686 |
| Cluster-2231.5675 | K13953 | ADH1_7; alcohol dehydrogenase 1/7 [EC:[1.1.1.1](https://www.kegg.jp/entry/1.1.1.1)] | -2.0232 |
| Cluster-2231.3683 | K13953 | ADH1_7; alcohol dehydrogenase 1/7 [EC:[1.1.1.1](https://www.kegg.jp/entry/1.1.1.1)] | -1.89 |
| Cluster-2231.4708 | K00511 | SQLE, ERG1; squalene monooxygenase [EC:[1.14.14.17](https://www.kegg.jp/entry/1.14.14.17)] | -1.3828 |
| Cluster-2045.0 | K01852 | LSS, ERG7; lanosterol synthase [EC:[5.4.99.7](https://www.kegg.jp/entry/5.4.99.7)] | -6.7669 |
| Cluster-2231.4402 | K05917 | CYP51; sterol 14alpha-demethylase [EC:[1.14.14.154](https://www.kegg.jp/entry/1.14.14.154) [1.14.15.36](https://www.kegg.jp/entry/1.14.15.36)] | -2.0975 |
| Cluster-2231.4471 | K09827 | ERG27; 3-keto steroid reductase [EC:[1.1.1.270](https://www.kegg.jp/entry/1.1.1.270)] | -1.7867 |
| Cluster-2231.8733 | K14674 | LIPA; lysosomal acid lipase/cholesteryl ester hydrolase [EC:[3.1.1.13](https://www.kegg.jp/entry/3.1.1.13)] | 3.8645 |
| Cluster-2231.7343 | K00559 | SMT1, ERG6; sterol 24-C-methyltransferase [EC:[2.1.1.41](https://www.kegg.jp/entry/2.1.1.41)] | 2.0451 |
| Cluster-2231.7522 | K09829 | ERG2; C-8 sterol isomerase [EC:5.-.-.-] | 1.7401 |
| Cluster-2231.2785 | K09831 | ERG5, CYP61A; sterol 22-desaturase [EC:[1.14.19.41](https://www.kegg.jp/entry/1.14.19.41)] | -2.3453 |
| Cluster-2231.5025 | K00223 | ERG4; Delta24(24(1))-sterol reductase [EC:[1.3.1.71](https://www.kegg.jp/entry/1.3.1.71)] | 2.2352 |
| Cluster-2231.4402 | K05917 | CYP51; sterol 14alpha-demethylase [EC:[1.14.14.154](https://www.kegg.jp/entry/1.14.14.154) [1.14.15.36](https://www.kegg.jp/entry/1.14.15.36)] | -2.0957 |
| Glycerophospholipid metabolism | | | |
| Cluster-2231.302 | K06123 | AYR1; 1-acylglycerone phosphate reductase [EC:[1.1.1.101](https://www.kegg.jp/entry/1.1.1.101)] | -6.2015 |
| Cluster-2231.4312 | K13509 | AGPAT1_2; lysophosphatidate acyltransferase [EC:[2.3.1.51](https://www.kegg.jp/entry/2.3.1.51)] | -1.3345 |
| Cluster-2231.3972 | K16368 | DGK1; diacylglycerol kinase (CTP) [EC:[2.7.1.174](https://www.kegg.jp/entry/2.7.1.174)] | -1.0226 |
| Cluster-2231.4041 | K01613 | psd, PISD; phosphatidylserine decarboxylase [EC:[4.1.1.65](https://www.kegg.jp/entry/4.1.1.65)] | -2.7021 |
| Cluster-2231.2996 | K01613 | psd, PISD; phosphatidylserine decarboxylase [EC:[4.1.1.65](https://www.kegg.jp/entry/4.1.1.65)] | -2.5617 |
| Cluster-2231.8645 | K14674 | PLA2G, SPLA2; secretory phospholipase A2 [EC:[3.1.1.4](https://www.kegg.jp/entry/3.1.1.4)] | 3.8645 |
| Cluster-2231.4322 | K13621 | BTA1; betaine lipid synthase | -3.3826 |
| Cluster-2231.8733 | K14674 | PLA2G, SPLA2; secretory phospholipase A2 [EC:[3.1.1.4](https://www.kegg.jp/entry/3.1.1.4)] | 3.8645 |
| Cluster-2231.4217 | K13333 | LYPLA1; lysophospholipase I [EC:[3.1.1.5](https://www.kegg.jp/entry/3.1.1.5)] | -2.555 |
| Cluster-2231.1803 | K00967 | PCYT2; ethanolamine-phosphate cytidylyltransferase [EC:[2.7.7.14](https://www.kegg.jp/entry/2.7.7.14)] | 2.2492 |
| Cluster-2231.9404 | K01126 | GDE1; glycerophosphodiester phosphodiesterase [EC:[3.1.4.46](https://www.kegg.jp/entry/3.1.4.46)] | 3.0236 |
| Cluster-2231.5689 | K00866 | CKI1; choline kinase [EC:[2.7.1.32](https://www.kegg.jp/entry/2.7.1.32)] | 2.6583 |
| Fatty acid degradation | | | |
| Cluster-2231.6634 | K00232 | E1.3.3.6, ACOX1, ACOX3; acyl-CoA oxidase [EC:[1.3.3.6](https://www.kegg.jp/entry/1.3.3.6)] | 3.9072 |
| Cluster-2231.1617 | K00626 | ACAT, atoB; acetyl-CoA C-acetyltransferase [EC:[2.3.1.9](https://www.kegg.jp/entry/2.3.1.9)] | 2.2887 |
| Cluster-2231.6349 | K14338 | XANG; xanthocillin biosynthesis cytochrome P450 monooxygenase [EC:1.14.-.-] | -3.5946 |

Note： “T” and “C” represent treated groups and untreated groups, respectively.

Supplementary Table 2 summary of a part of major genes related to genetic information processing affected in *F. solani* exposed to 1,8-cineole

| ID | KEGG pathway | Enzyme | Fold change  (T/C) |
| --- | --- | --- | --- |
| RNA polymeraseRNA | | | |
| Cluster-2231.2734 | K03020 | RPAC2, RPC19, POLR1D;DNA-directed RNA polymerases I and III subunit RPAC2 DNA | -1.1742 |
| Cluster-2231.4987 | K03023 | RPC3, POLR3C; DNA-directed RNA polymerase III subunit RPC3 | -1.7383 |
| Spliceosome |  |  |  |
| Cluster-2231.4484 | K12820 | DHX15, PRP43; pre-mRNA-splicing factor ATP-dependent RNA helicase DHX15/PRP43 | -1.2159 |
| Cluster-2231.3529 | K12813 | DHX16; pre-mRNA-splicing factor ATP-dependent RNA helicase DHX16 [EC:[3.6.4.13](https://www.kegg.jp/entry/3.6.4.13)] | -1.7181 |
| Aminoacyl-tRNA biosynthesis | | | |
| Cluster-2231.3429 | K01886 | QARS, glnS; glutaminyl-tRNA synthetase [EC:[6.1.1.18](https://www.kegg.jp/entry/6.1.1.18)] | -1.1109 |
| Cluster-2231.3330 | K01876 | DARS2, asps; aspartyl-tRNA synthetase [EC:[6.1.1.12](https://www.kegg.jp/entry/6.1.1.12)] | -1.1287 |
| Cluster-2231.4175 | K01893 | NARS, asnS; asparaginyl-tRNA synthetase [EC:[6.1.1.22](https://www.kegg.jp/entry/6.1.1.22)] | -1.0266 |
| Cluster-2231.4381 | K01880 | glyQ; glycyl-tRNA synthetase alpha chain [EC:[6.1.1.14](https://www.kegg.jp/entry/6.1.1.14)] | -1.1244 |
| Cluster-2231.3190 | K01883 | CARS, cysS; cysteinyl-tRNA synthetase [EC:[6.1.1.16](https://www.kegg.jp/entry/6.1.1.16)] | -1.2623 |
| mRNA surveillance pathway mRNA | | | |
| Cluster-2231.6506 | K14405 | FIP1L1, FIP1; pre-mRNA 3'-end-processing factor FIP1 | 1.6803 |
| Cluster-2231.2610 | K15542 | PFS2; polyadenylation factor subunit 2 | 1.3055 |
| Cluster-2231.8809 | K14326 | UPF1, RENT1; regulator of nonsense transcripts 1 [EC:[3.6.4.13](https://www.kegg.jp/entry/3.6.4.13) [5.6.2.3](https://www.kegg.jp/entry/5.6.2.3)] | 2.5882 |
| Protein processing in endoplasmic reticulum | | | |
| Cluster-2231.6207 | K01228 | MOGS; mannosyl-oligosaccharide glucosidase [EC:[3.2.1.106](https://www.kegg.jp/entry/3.2.1.106)] | 1.163 |
| Cluster-2231.8177 | K05546 | GANAB; mannosyl-oligosaccharide alpha-1,3-glucosidase [EC:[3.2.1.207](https://www.kegg.jp/entry/3.2.1.207)] | 2.6231 |
| Cluster-2231.5387 | K14016 | UFD1; ubiquitin fusion degradation protein 1 | 1.1499 |
| Cluster-2231.4630 | K10601 | SYVN1, HRD1; E3 ubiquitin-protein ligase synoviolin [EC:[2.3.2.27](https://www.kegg.jp/entry/2.3.2.27)] | 1.0578 |
| Cluster-2231.2326 | K14026 | SEL1, SEL1L; SEL1 protein | 1.3303 |
| Ubiquitin mediated proteolysis | | | |
| Cluster-2231.7989 | K10684 | UBLE1A, SAE1; ubiquitin-like 1-activating enzyme E1 A [EC:[6.2.1.45](https://www.kegg.jp/entry/6.2.1.45)] | 1.0026 |
| Cluster-2231.3120 | K10589 | UBE3C; biquitin-protein ligase E3 C [EC:[2.3.2.26](https://www.kegg.jp/entry/2.3.2.26)] | 1.2957 |
| Cluster-2231.6227 | K03363 | CDC20; cell division cycle 20, cofactor of APC complex | 3.4001 |
| Cluster-2231.8274 | K03357 | APC10, ANAPC10, DOC1; anaphase-promoting complex subunit 10 | 1.2063 |
| RNA degradation | | | |
| Cluster-2231.6211 | K01689 | RRP42, EXOSC7; exosome complex component RRP42 | -6.2432 |
| Cluster-2231.3959 |  |  | -1.4542 |
| Cluster-2231.2129 | K11600 | RRP41, EXOSC4, SKI6; exosome complex component RRP41 | 1.4657 |
| Cluster-2231.1013 | K12581 | NOT7_8, CAF1, POP2; CCR4-NOT transcription complex subunit 7/8 | -4.3719 |
| Cluster-2231.2215 | K12620 | LSM1; 6 snRNA-associated Sm-like protein LSm1 | 1.6501 |
| Cluster-2231.6211 | K01689 | ENO, eno; enolase [EC:[4.2.1.11](https://www.kegg.jp/entry/4.2.1.11)] | -6.2432 |
| Cluster-2231.3959 |  |  | -1.4542 |
| Cluster-2231.3932 | K04043 | dnaK, HSPA9; molecular chaperone DnaK | 1.5415 |
| DNA replication |  |  |  |
| Cluster-2231.1113 | K10742 | DNA2; DNA replication ATP-dependent helicase/nuclease Dna2 [EC:[5.6.2.3](https://www.kegg.jp/entry/5.6.2.3) 3.1.-.-] | 1.1677 |
| Cluster-2231.924 | K10747 | LIG1; DNA ligase 1 [EC:[6.5.1.1](https://www.kegg.jp/entry/6.5.1.1) [6.5.1.6](https://www.kegg.jp/entry/6.5.1.6) [6.5.1.7](https://www.kegg.jp/entry/6.5.1.7)] | 1.1827 |
| Cluster-2231.1706 | K02327 | POLD1; DNA polymerase delta subunit 1 [EC:[2.7.7.7](https://www.kegg.jp/entry/2.7.7.7)] | 1.2287 |
| Cluster-2231.7237 | K03504 | POLD3; DNA polymerase delta subunit 3 | 1.389 |
| Base excision repair |  |  |  |
| Cluster-2231.6902 | K10771 | APEX1; AP endonuclease 1 [EC:[4.2.99.18](https://www.kegg.jp/entry/4.2.99.18)] | 1.3562 |
| Cluster-2231.4651 | K02324 | POLE; DNA polymerase epsilon subunit 1 [EC:[2.7.7.7](https://www.kegg.jp/entry/2.7.7.7)] | -1.1781 |
| Cluster-2231.924 | K10747 | LIG1; DNA ligase 1 [EC:[6.5.1.1](https://www.kegg.jp/entry/6.5.1.1) [6.5.1.6](https://www.kegg.jp/entry/6.5.1.6) [6.5.1.7](https://www.kegg.jp/entry/6.5.1.7)] | 1.1827 |
| Nucleotide excision repair | | | |
| Cluster-2231.4651 | K02324 | POLE; DNA polymerase epsilon subunit 1 [EC:[2.7.7.7](https://www.kegg.jp/entry/2.7.7.7)] | -1.1781 |
| Cluster-2231.924 | K10747 | LIG1; DNA ligase 1 [EC:[6.5.1.1](https://www.kegg.jp/entry/6.5.1.1) [6.5.1.6](https://www.kegg.jp/entry/6.5.1.6) [6.5.1.7](https://www.kegg.jp/entry/6.5.1.7)] | 1.1827 |
| Mismatch repair | | | |
| Cluster-2231.958 | K08737 | PMS2; DNA mismatch repair protein PMS2 | 1.5423 |
| Cluster-2231.4010 | K08735 | MSH2; DNA mismatch repair protein MSH2 | -3.1511 |
| Cluster-2231.4511 | K08739 | MLH3; DNA mismatch repair protein MLH3 | 2.1265 |
| Cluster-2231.924 | K10747 | LIG1; DNA ligase 1 [EC:[6.5.1.1](https://www.kegg.jp/entry/6.5.1.1) [6.5.1.6](https://www.kegg.jp/entry/6.5.1.6) [6.5.1.7](https://www.kegg.jp/entry/6.5.1.7)] | 1.1827 |

Note： “T” and “C” represent treated groups and untreated groups, respectively.
